# The Routes of Emergence of Life from LUCA during the RNA and Viral World: A Conspectus

**DOI:** 10.3390/life5021445

**Published:** 2015-06-05

**Authors:** Sohan Jheeta

**Affiliations:** Network of Researchers on Horizontal Gene Transfer and the Last Universal Cellular Ancestor (NoR HGT & LUCA †), 1 Scott Hall Crescent, Leeds LS7 3RB, UK; E-Mail: sohan7@ntlworld.com; Tel.: +44-0113-2628767

**Keywords:** horizontal gene transfer, HGT, LUCA, transduction, transformation, RNA, virus, RNA-dependent RNA polymerases, origin of life

## Abstract

How did life emerge on Earth? The aim of the Network of Researchers on Horizontal Gene Transfer and the Last Universal Cellular Ancestor (NoR HGT & LUCA) is to understand how the genetics of LUCAs were reorganised prior to the advent of the three domains of life. This paper reports the research of eminent scientists who have come together within the network and are making significant contributions to the wider knowledge base surrounding this, one of science’s remaining mysteries. I also report on their relevance in relation to LUCAs and life’s origins, as well as ask a question: what next?

## 1. Introduction

It is perplexing, the origin of life. This is because understanding the processes of life’s origin is mind-bogglingly complex. It has been over 60 years since Stanley Miller carried out his world famous “electric discharge” experiment, and the newspapers subsequently heralded “life in a test tube” after he had succeeded in making amino acids from “electrified” primordial gases [1]. To date, we have not even dented the metaphoric surface of the mysteries of the origin of life; for example, we are still grappling with the question of which came first, metabolism, genetics or vesicles? How else could the details of life’s origins be unearthed? One way of augmenting the knowledge base and thereby shedding more light on this issue is to study the relevance of horizontal gene transfer during the evolution of the pre-last universal cellular ancestor, up to the point of emergence of the two domains of life, namely Archaea and Bacteria, with Eukarya being the chimera of the first two [2,3]. The Network of Researchers on Horizontal Gene Transfer and the Last Universal Cellular Ancestor’s (NoR HGT & LUCA) focus is on studying the role played by HGT and its fine tuning of LUCA genetics. Since it is “generally” agreed that there was a living organism with a genetic system based entirely on RNA [4,5] in the “shape” of a LUCA, our network’s aim is to “peel” back the “metaphoric onion of LUCA” from the first emergence of life to the point of preRNA chemistry [6]. The 2014 NoR HGT & LUCA meeting was entitled: “the routes of emergence of life from LUCA during the RNA and viral world” and was held at the University of Leeds, UK, October 2014. The meeting brought together eminent researchers from different disciplines to slowly add “meat onto LUCA’s metaphoric bone”. This paper gives a synopsis of the meeting with emphasis on the correlation between the featured research and LUCA.

## 2. Horizontal Gene Transfer

The more widely-recognised modes of HGT include transduction and transformation, and these are thought to be the result of Darwinian evolution. If this is the case, then how did HGT occur during the genetic evolution of pre-LUCAs in the absence of Darwinian evolution (scientists, including Forterre [5] and Poole [7], postulated that before LUCA, there was a pre-LUCA)? Lightning is one possible non-biochemical natural mechanism put forward by Kotnik [8,9]; in the presence of a sufficiently strong electric field, the lipid bilayer of the membrane becomes permeable due to the presence of “aqueous holes” traversing the membranes. It is through these holes that many molecules, including DNA and RNA, can pass unhindered into cells [10]; could this mode of gene transfer have aided the tweaking of early LUCA’s genetics? Kotnik asserts that this channel of HGT may well be used by contemporary microorganisms where the three main modes of HGT are absent. I would also extrapolate that this type of gene transfer would have been prevalent during the pre-LUCA epoch. Tuller [11,12,13] reviews the mechanisms by which bias contributed to the success of HGT and points out the ramifications pertaining to the codon bias similarities, demonstrating that organisms with a similar codon bias tend to be involved in HGTs, aiding the development of LUCAs from pre-LUCAs; whereas dissimilarity in codon bias seems to be a central barrier to HGT. 

There is some evidence in favour of a LUCA being present at the dawn of the emergence of life [14]. Forterre [15] lays the ground for what was expected of LUCA, including hypothesising the presence of “RNA-cells” and the intervention of viruses, which gave these cells DNA and DNA replication machinery, for example, via HGT; DNA being a more stable molecule, whose sole function is to carry chemically-coded information. Horizontal transfer of DNA, RNA, proteins and lipids can occur between cells involving membrane vesicle transfers (MVTs) [16]. Such mechanisms, if present in a LUCA, would have been instrumental in reorganising traits and augmenting new features. Alternate transfer mechanisms, gene transfer agents (GTAs) are phage-like particles, which transfer DNA. These GTAs continue to be maintained in both Archaea and Bacteria (e.g., RcGTA, produced by Rhodobacter capsulatus, is retained in bacteria), which raises interesting questions as to the origins of phages and the evolutionary importance of HGT [17]. It is to be noted that both MVTs and GTAs are modes of horizontal transfer, which are akin to transduction. Transduction can also occur between unrelated viruses, albeit a less common phenomenon. Presumably, this phenomenon would have aided the successful development of viral genetic diversity, as well as the modification of LUCAs, especially during the advent of DNA, into the three domains of life we observe on Earth [18].

HGT can also be carried out by transformation, where there is active take-up of heterologous DNA from the environment by recipient competent cells, followed by recombination or replication. Overballe-Petersen *et al*. [19] elaborates on this mechanism and states that short, ancient and often damaged DNAs could be taken up by the extant cells from the environment. Such DNAs have evolutionary implications for microbes in that ancient genes can be resuscitated, giving microbes new traits, thus indicating that HGT could be seen as an evolutionary strategy supporting the evolution of sexual reproduction.

## 3. RNA-Dependent RNA Polymerases

RNA-dependent RNA polymerases (RdRps) are enzymes that bring about replication of RNA from an RNA template, and these enzymes are part and parcel of the genomes of all RNA-containing viruses, with no DNA involvement at any stage. More importantly, they are prone to introducing mutations, especially during viral genome replication, thus causing the virus to evolve quickly. To study the rare biological events, a “single molecule technique” is an amazing toolbox that could be deployed; a particularly relevant process is the mechanism of error incorporation by RNA polymerase involved in genome replication, which is the basis of evolution. Approaches, such as stop flow assays, have been developed and have shed light on many sub-steps in nucleotide incorporation. However, they struggle to distinguish low probability events from the bulk, as error incorporation. On the other hand, single molecule techniques were not parallelised enough to reach the necessary statistic to characterise events happening only one in a thousand to ten thousand. Therefore, Dulin *et al.* [20] have developed a highly parallelised magnetic tweezers assay, which allows one to follow hundreds of RNA or DNA templates at once and in real-time, with a sub-nanometre resolution [20]. This method has been applied to error-prone RdRPs, in order to characterise their error incorporation mechanism [21]. Such techniques could also be used to understand LUCAs’ genome via studying viral genetics. RdRps are also involved in defending eukaryotes against attack by transposable elements. In general, PiWi interacting small RNAs (piRNAs) are crucial in protecting eukaryotes against transposable elements, but when piRNAs are absent, RdRps are deployed [22]. The presence of RdRps would have been important from the vantage point of affording LUCAs protection whilst their genetics were modified, as it is believed that at the dawn of the emergence of life, LUCAs were fully functional organisms that displayed cellular life.

## 4. On the Development of RNA

The emergence of life depended on the chemical evolution of RNA and its subsequent ability to carry chemical information, to perform catalytic activity, to self-replicate and to be subjected to mutation. The latter event allowed the molecule to evolve according to the changing environmental conditions in the form of selection pressures. Krishnamurthy [23] asserts that the optimal precursor(s) of RNAs would have been selected from a library of structures (a combination of various backbones, linkers, nucleobases and connectives) based on their prime functions and also draws attention to the relationship between the structure and the function of a molecule. This position seems to be reinforced by Powner *et al.* [24,25], demonstrating the first chemical steps towards a divergent pyrimidine and purine ribonucleotide synthesis. The ambiguity arising from 5'–2' *versus* 5'–3' phosphodiester bonds during preRNA chemistry and, in the absence of sophisticated enzymes in general, with the eventual formation of 5'–3' phosphodiester bonds, results in an important step forward for the canonical position of RNA as a self-replicator [26]. In order to gain greater understanding of the early efficient self-replication mechanism, some insight into the engineering and evolution of RNA polymerase ribozymes, as well as the potential role that structured media, such as the eutectic phase of water ice, may have played in the emergence of RNA self-replication is posited. 

**Figure 1:**
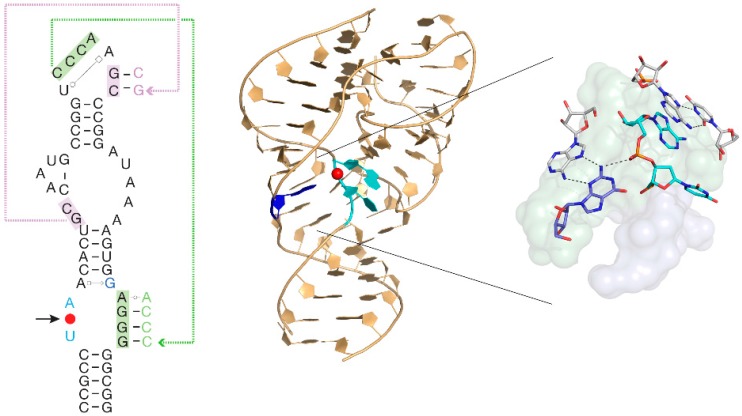
The twister ribozyme. The sequence and secondary structure of the twister ribozyme (**left**) and the crystal structure (**centre**) with the active centre expanded (**right**). The red sphere in the sequence and structure is the scissile phosphate in the cleavage reaction.

During the LUCA epoch in particular, ribozymes were very important catalysts, which modified RNAs by splicing them [27,28]. Twister ribozymes are widespread in many genomes, located within non-coding RNA sequences, and are strongly conserved and expressed within the cell. The RNA adopts a novel compact fold based on a unique reversed, double-pseudoknot structure, with the scissile phosphate at its centre. This is shown as the red spot in Figure 1, revealing the position of the active centre of the ribozyme. Like other nucleolytic ribozymes, twister employs general acid-base catalysis to accelerate its cleavage reaction about a million fold, involving participating guanine and adenine nucleobases [29]. Moreover, this ribozyme is a molecular fossil, which may have been present at the dawn of the emergence of life on Earth.

What is the origin of such ribozymes? In a new hypothesis entitled: “tRNA core hypothesis: a new model for the origin of the biological system” put forward by Farias *et al.* [30], it is essentially purported that proto-tRNA came first and that it initially formed both the proto-mRNA and proto-rRNA; together, these then synthesised a proto-translational machine for making initial rudimentary proteins; proteins being efficient enzymes, thus accelerating the development of LUCAs in the form of replicators.

## 5. Alternative Hypotheses

The lipid-world hypothesis is a direct alternative to that of an RNA world, claiming that compositional assemblies, such as vesicles with multiple amphiphile types, can replicate and undergo evolution [31]. A computer model, entitled graded autocatalysis replication domain (GRAD), was deployed to study the properties of such compositional assemblies, and these simulations indicated that GRAD assemblies can transmit compositional information through catalysed homeostatic growth followed by random fission [32], unlike the template-based replication of polymeric strands of DNA or RNA. The key to GRAD dynamics are composomes (spontaneously-forming replication-prone compositional states), which led to the formation of compotypes in the context of a “lipid or GRAD world”. Such compotypes displayed Darwinian evolution [33], and they behaved like quasi-species [34], thus suggesting a progression from a random mixture of primordial soup to replicating and evolving entities. 

A further step would have been the formation of evolvable vesicles made from non-living molecules, which contained strands of RNA. These initial RNAs replicated through the translation of encoded RNA replicase for 600 generations, and during such replications, mutations in the initial RNA genomes were introduced spontaneously. This soon led to the formation of highly “replicable parasitic (selfish) mutants”; with continued replication, the initial RNA genome gradually reinforced its interaction with the translated replicase, thereby gaining a competitive edge against the mutant RNAs. This showed the presence of Darwinian evolution that probably began during the “RNA world”, *i.e.*, the “LUCA world” [35]. 

The presence of these replicators is speculated, and it is also asserted that, according to the literature, nucleotides could spontaneously form and even join RNA oligomers in high concentrations of formamide [36]; such an environment would be found in the vapours arising from geothermal systems [37]. Formamide’s additional roles in the origin of life have been studied by Saladino *et al.* [38] and Pino *et al.* [39]. Their initial focus was on the spontaneous generation of metabolically-important compounds observed in biology today—from hydrogen cyanide (HCN) through to the formation of formamide (NH_2_COH) and then on to a whole host of other “living” compounds. This included the formation of carboxylic and amino acids (metabolism first hypothesis), as well as the synthesis of 3',5'-cyclic nucleotides (e.g., cAMP), which formed RNA polymers, such as ribozyme-like catalysts (genetics first hypothesis). The eventual result was that there was no bifurcation of metabolism and genetics, but that both were inter-mingling to form the biology as we know it today; such conclusions were reached via additional experimental, theoretical and computer simulation studies [38,39].

Other biologically-necessary compounds are cofactors, of which one of the most important is nicotinamide adenine dinucleotide (NAD) [40]. NAD is involved in many redox reactions, as well as being involved in several biological processes as a substrate for the signalling of the enzymes that consume it, and its central role in biology is ascertained by its capability of self-synthesis in an autocatalytic pathway. The central tenet is that extant precursors of NAD, such as NMN (nicotinamide mononucleotide) or other pyridine-based dinucleotides, would effectively perform the same, at least co-enzymatic, job. The NAD cofactor is thought to have predated the other pyridine derivatives (e.g., guanine nucleotide) that have been present during the “metabolic world” and, as such, would have been involved with the formation of proto-enzymes during the dawn of the RNA world.

## 6. Photosynthesis and Eukaryotes

The alkaline hydrothermal vent hypothesis predicts that life arose in the watery environment on the seabed, and that life’s reactions were taking place in tiny clay bubbles, formed during the escape of gas from the mantle, with energy being derived from geothermal sources. In order for life to break out of these bubbles and become an independent, free floating entity, it became necessary for the electro-chemical potential and ionic concentration gradient across cellular boundaries to be regulated and maintained (homeostasis). Such an arrangement would generate the proton motive forces needed for metabolism and active transport, *etc.* Allen [41] addresses this issue by hypothesising that the initial autotrophic process was of an anoxygenic type, meaning that oxygenic photosynthesis arose later. Furthermore, the oxygenic photosynthesis resulted from the failure of a redox switch (encoded in the genome) that maintains redox homeostasis during anoxygenic photosynthesis. Still on the theme of photosynthesis, the evolution of the photosynthetic apparatus in the eukaryote *Paulinella chromatophora* has been investigated with the objective being to gain insight into how the genetic repertoire of *P. chromatophora* evolves from the merger of two physiologically- and genetically-different cells that allowed the host cell (an ancient heterotrophic protist) to control and manipulate the chromatophore (a cyanobacterium) according to its needs [42,43,44]. Essentially, the relationship is not too dissimilar from the one between the symbionts that formed a modern eukaryote, that is, between the “ancient bacterial cell” and the “mitochondrial-equivalent cell”, and this research should yield more interesting results by way of possibly explaining the origin of eukaryotes.

## 7. What Next?

To fully understand the evolution of life from simple non-living chemicals and chemical reactions to the formation of three-dimensional life forms is a challenging matter, encompassing multi-faceted research and investigation into diverse areas of both chemistry and physics. The research pinpoints crucial areas in life’s early development, but intriguing gaps still remain. Key indicators in the report, such as the importance of lightning during HGT, the role played by eutectics in icy water that led to the emergence of RNA and the chemical evolution of viruses from early prehistoric chemistry, *etc.*, can create a good foundation upon which to build. Other significant questions remain: what environmental conditions were needed? In what location did life first form? Is RNA chemistry a red herring? We have no definitive answers; however, as can be seen from the scope and quality of the research by such a high calibre group of scientists, there is a willingness to develop further insights into this exceptionally stimulating scientific area and then disseminate the knowledge amongst the widest audience possible. 
